# CE–MS for anionic metabolic profiling: An overview of methodological developments

**DOI:** 10.1002/elps.201900115

**Published:** 2019-06-04

**Authors:** Marlien van Mever, Thomas Hankemeier, Rawi Ramautar

**Affiliations:** ^1^ Biomedical Microscale Analytics Leiden Academic Centre for Drug Research Leiden University Leiden The Netherlands; ^2^ Analytical BioSciences & Metabolomics Leiden Academic Centre for Drug Research Leiden University Leiden The Netherlands

**Keywords:** Anionic metabolic profiling, Applications, Mass Spectrometry, Metabolomics, Methodological Developments

## Abstract

The efficient profiling of highly polar and charged metabolites in biological samples remains a huge analytical challenge in metabolomics. Over the last decade, new analytical techniques have been developed for the selective and sensitive analysis of polar ionogenic compounds in various matrices. Still, the analysis of such compounds, notably for acidic ionogenic metabolites, remains a challenging endeavor, even more when the available sample size becomes an issue for the total analytical workflow. In this paper, we give an overview of the possibilities of capillary electrophoresis‐mass spectrometry (CE–MS) for anionic metabolic profiling by focusing on main methodological developments. Attention is paid to the development of improved separation conditions and new interfacing designs in CE–MS for anionic metabolic profiling. A complete overview of all CE–MS‐based methods developed for this purpose is provided in table format (Table 1) which includes information on sample type, separation conditions, mass analyzer and limits of detection (LODs). Selected applications are discussed to show the utility of CE–MS for anionic metabolic profiling, especially for small‐volume biological samples. On the basis of the examination of the reported literature in this specific field, we conclude that there is still room for the design of a highly sensitive and reliable CE–MS method for anionic metabolic profiling. A rigorous validation and the availability of standard operating procedures would be highly favorable in order to make CE–MS an alternative, viable analytical technique for metabolomics.

**Abbreviations**
FA: fatty acid
HILIC: hydrophilic interaction liquid chromatography
IPR: ion‐pair reagent
MSI: multi‐segment injection
PIESI: paired ion electrospray ionization
SL: sheath‐liquid

## 1 Introduction

Metabolomics is considered within the field of analytical chemistry a well‐accepted analytical approach for the global profiling of metabolites, i.e., small (endogenous) molecules with a molecular weight below 1500 Da. Currently, the Human Metabolome Database contains more than 100 000 metabolite entries with a wide dynamic concentration range, i.e., from mM to the pM‐level 1. A major part of these metabolite entries consists of lipids and exogenous compounds derived from nutrients and drugs. The metabolome is affected by both internal and external factors/stimuli and, therefore, directly reflects the underlying biochemical activity and status of the biological system in question. Metabolic profiles may provide a wealth of information which can be used for disease prediction, disease progression, and treatment outcome 2, 3.

Nowadays, analytical techniques such as NMR spectroscopy, LC–MS, and GC–MS are generally used for metabolic profiling studies 4, 5, 6. CE–MS has emerged as a strong analytical tool for the profiling of polar and charged metabolites, such as phosphorylated sugars, organic acids, amino acids, and nucleotides, since its separation mechanism is based on charge‐to‐size ratios, thereby providing complementary information to other separation techniques 7. Like CE–MS, hydrophilic interaction liquid chromatography (HILIC)–MS also emerged as a powerful analytical tool for the profiling of (highly) polar metabolites. Recent studies have indicated that both analytical techniques provide complementary metabolic information and in that context the use of both approaches is preferably required in order to get a full picture of the polar and charged compounds present in a given biological sample 8, 9, 10. It would also be interesting to compare CE–MS with zwitter‐ionic HILIC columns and recently developed ion‐exchange LC systems for metabolic profiling studies. Ion‐pair reversed‐phase LC–MS has also been employed for the profiling of polar and charged metabolites 11, 12. However, the use of ion‐pair agents in LC–MS may result in severe ion suppression and may contaminate the ion source and ion optics. In addition, ion‐pair agents may contribute to column instability and increased re‐equilibration time.

The first research where CE–MS was used for global metabolic profiling of biological samples was performed by Soga and co‐workers 13. In this work, a bare fused‐silica capillary and acidic separation conditions were used for the analysis of cationic metabolites, performing CE–MS in positive ionization mode. In general, this CE–MS method has been used now by various research groups as it provides acceptable performance metrics for the profiling of cationic metabolites 14, 15, 16, 17, 18, 19, 20. However, in order to attain full coverage of ionogenic metabolites, both cationic and anionic metabolites need to be analyzed by CE–MS. However, for anionic metabolic profiling the number of studies with CE–MS reported in the literature is far less than the number of studies involving cationic metabolic profiling. Soga et al. studied the anionic metabolic profile of extracts from *Bacillus subtilis* cells by CE–MS using a cationic polymer‐coated capillary and weakly alkaline ammonia buffers, employing reversed CE polarity and negative ionization mode 21, 22. Büscher et al. performed a cross‐platform study for metabolic profiling of yeast extracts, and indicated CE as the least suitable platform for analyzing biological samples as it lacked the required robustness 23. On the other hand, the CE–MS method at low‐pH separation conditions employing a fused‐silica capillary could be used in a robust way for cationic metabolic profiling of a yeast extract, and observed matrix effects were significantly lower as compared to the other chromatographic methods evaluated in this work. In agreement with the work of Büscher et al., Soga et al. demonstrated in another work that the long‐term stability of the CE–MS method using a cationic‐coated capillary for anionic metabolic profiling was relatively poor 24. Authors found that the stability issue with the CE–MS method for anionic metabolic profiling appeared to be caused by corrosion of the stainless steel ESI needle when employing reversed CE polarity and negative ionization mode conditions. To overcome this issue, a platinum sprayer needle was used for CE–MS analysis in reversed polarity mode 24, although the platinum sprayer is not necessary for anionic metabolic profiling when applying normal CE polarity at high‐pH separation conditions. Because of these stability issues together with lower sensitivity using negative ionization mode detection, the perception had risen that CE–MS was not suitable for global metabolic profiling, especially when compared to other chromatographic techniques such as LC–MS and GC–MS.

It is clear that the development of a robust and sensitive CE–MS approach for anionic metabolic profiling requires special attention. The improvement of CE–MS separation conditions and recent developments in interfacing designs, such as the sheathless porous‐tip interface and the use of modified sheath‐liquid (SL) interfaces, show great potential for sensitivity enhancement of profiling anionic metabolites. Therefore, in this review an overview of CE–MS approaches for anionic metabolic profiling is provided, covering the literature published between May 2002 and December 2018. In that context, the current work can be regarded as an important (complementary) addition to our previous CE–MS‐based metabolomics reviews 25, 26, 27, 28, 29, 30, which we provide bi‐annually for Electrophoresis and in which the usefulness and developments of CE–MS approaches for anionic metabolic profiling were not considered in detail so far. Major methodological developments that led to the improvement of the reproducibility and sensitivity of CE–MS for anionic metabolic profiling are considered and representative examples in various application fields are highlighted.

## 2 Methodological developments

In this section, we will pay attention to CE–MS approaches that have been developed for the profiling of anionic metabolites, with a main focus on improvement of detection sensitivity (i.e., metabolic coverage) and reproducibility. A comprehensive overview of CE–MS methods developed for the profiling of anionic metabolites in the time period from May 2002 until December 2018 is provided in Table 1. Only those studies are included in Table 1 which report the development of new CE–MS approaches for anionic metabolic profiling. For a complete overview of classical CE–MS employing a standard co‐axial SL interface at high‐pH separation conditions and MS detection in negative ion mode, i.e., the approach also used by the company Human Metabolome Technologies, we refer to especially the tables of our previous reviews 25, 26, 27, 28, 29, 30.

**Table 1:** Overview of CE–MS‐based anionic metabolic profiling studies reported between May 2002 and December 2018

Application field	Sample matrix	BGE	Capillary	MS analyzer	LODa	Notes	Ref.
Biomedical	Liver and serum extracts of acetaminophen‐treated mice	50 mM ammonium acetate (pH 8.5)	Cationic polymer: SMILE(+) capillary	TOF	ns.		54
	Mouse liver extracts	50 mM ammonium acetate (pH 8.5)	Cationic polymer: COSMO(+) capillary	TOF	0.03–0.87 µM	Platinum ESI needle; internal standards for quantification	24
	Human urine, rat urine	25 mM triethylamine (pH 11.7)	Fused‐silica capillary	(Q‐)TOF	0.7–9.1 µM		31, 55
	Human urine	50 mM ammonium bicarbonate (pH 9.5)	Fused‐silica capillary	TOF	0.4 µM		56
	Human serum	5 mM ammonium acetate (pH 10.8)	Fused‐silica capillary	QqQ	0.05–0.81 µM	Home‐made sheathless interface	57
	Human Urine	5 mM ammonium acetate in ACN/MeOH 80:20 (v/v)	Fused‐silica capillary with a porous tip	Quadrupole	5 ng/mL	Non‐aqueous capillary electrophoresis (NACE); porous tip sheathless interface	45
	Glioblastoma cells	10% acetic acid (pH 2.2)	Fused‐silica capillary with a porous tip	TOF	10–200 nM	Low pH BGE for anionic metabolic profiling; porous tip sheathless interface	39
	Human urine	50 mM ammonium bicarbonate (pH 8.5)	Fused‐silica capillary	TOF	ns.	Multi‐segment injection (MSI)‐CE–MS; hydrodynamic pressure gradient applied during separation	47
	Arixtra® and LMWH (Lovenox®)	10 mM ammonium acetate in 80% aqueous methanol (pH 7.5)	Fused‐silica capillary	LTQ Orbitrap	ns.		40
Application field	Sample matrix	BGE	Capillary	MS analyzer	LODa	Notes	Ref.
	HEK 294T cells	10% acetic acid (pH 2.2)	Fused‐silica capillary	TOF	ns.	Low pH BGE for anionic metabolic profiling; anionic metabolites detected in positive ion mode; no nebulizing gas applied; pressure of 30 mbar at the CE inlet	10
	Human plasma	50 mM ammonium acetate (pH 8.5)	Fused‐silica capillary	TOF	ns.	Internal standards for quantification	59
	Human plasma and serum	70% acetonitrile, 15% methanol, 10% H2O, and 5% isopropanol in ammonium acetate (pH 9.5)	Fused‐silica capillary	TOF	0.70 µM	Multi‐segment injection non‐aqueous capillary electrophoresis‐mass spectrometry; Internal standards for quantification	49
	*Aplysia californica* cells	20 mM ammonium bicarbonate (pH 8.2)	Fused‐silica capillary	Q‐TOF	5.5 nM	Custom‐built co‐axial sheath‐flow CE‐ESI interface; CE‐ESI emitter tip emerged in a N2 bath gas chamber	37
Microbial/plant	*Bacillus subtilis* cell extract	50 mM ammonium acetate (pH 9.0)	Cationic polymer: SMILE(+) capillary	IT	0.3–6.7 µM		22
	*Bacillus subtilis* cell extract	50 mM ammonium acetate (pH 7.5)	Neutral polymer: DB‐1 capillary	IT	0.4–3.7 µM	Pressure assisted CE (PACE)	21
	Anionic standards	50 mM trimethylamine acetate (pH 10.0)	Fused‐silica capillary	IT	ns.	Pressure assisted CE	58
	*E. coli* cell extracts	50 mM ammonium acetate (pH 7.5)	Fused‐silica capillary	IT	0.5–1.7 µM	Pressure assisted CE, silanol mask technique	59
Application field	Sample matrix	BGE	Capillary	MS analyzer	LODa	Notes	Ref.
	*Catharanthus roseus* cell extract	50 mM ammonium acetate (pH 9.0)	Sulfonated capillary: FunCap‐CE type S	TQ linear‐IT	0.1–8.8 µM	Multiple reaction monitoring (MRM)	60
	Yeast	150 mM ammonium hydrogencarbonate/formate (pH 6.0)	PEEK capillary	IT	ns.	Pressure assisted CE	61
	*E. coli* cell extracts	50 mM ammonium acetate (pH 8.7)	Fused‐silica capillary, coated with PolyE‐323	TOF	0.2–2 µM		62
	*E. coli* cell extracts	20 mM ammonium 2‐propanol (8:2 v/v) (pH 9.5)	Fused‐silica capillary with a porous junction	QIT	0.02–2.5 µM	Sheathless interface with home‐made porous junction	63
	Transgenic rice plants	50 mM ammonium acetate (pH 8.5); 50 mM ammonium acetate (pH 7.5)	Cationic polymer: SMILE(+) capillary; Neutral polymer: DB‐1 capillary	Quadrupole	0.3–6.7 µM; 0.4–3.7 µM		64
	Transgenic rice plants	20 mm ammonium acetate (pH 6.8)	Neutral polymer: DB‐WAX capillary	Quadrupole	ns.	Internal standards for quantification	65
	Pineapple leaves	1 M formic acid (pH 1.8)	Fused‐silica capillary	QqQ	0.5–10 µM	High‐speed sheath gas flow applied	34
	Moss extract (*Physcomitrella patens*)	50 mM ammonium formate (pH 8.0); 50 mM ammonium acetate (pH 10.0) containing 50% methanol	Fused‐silica capillary	IT	0.13–17 µM	Pressure assisted CE; Internal standards for quantification	66
Food/environmental	Ale	2 mM TMA and 5 mM Tris (pH 8.5)	Fused‐silica capillary	Quadrupole	0.05–0.1 µg/mL		67
	Apples, grapes, oranges, tomatoes	32 mM ammonium formate (pH 3.1)	Fused‐silica capillary	Quadrupole	0.1–3 µg/mL		68
	Apple juice	20 mM ammonium formate (pH 10)	Fused‐silica capillary	IT	1.1–3.5 µg/mL		69
	Orange juice and red wine	1 M formic acid, pH 2.4	PTH coated capillary	TOF	0.1–16.4 ppm		46
Application field	Sample matrix	BGE	Capillary	MS analyzer	LODa	Notes	Ref.
	Cheese and coffee samples	ammonium formate buffer containing 40% acetonitrile modifier	Fused‐silica capillary	TOF	0.13 to 2.88 mg/mL	Addition of ion pairing reagents as addition to the sheath liquid to detect anionic metabolites/ion pair complexes in positive ion mode	33

### 2.1 Improving sensitivity/metabolic coverage

A major issue observed in CE–MS using negative ionization mode are the relatively low metabolite responses in comparison to those in positive ionization mode. Kok et al. evaluated different BGE compositions and SL additives in order to enhance metabolite responses in CE–MS in negative ionization mode 31. It was found that the inclusion of triethylamine (TEA; pH 11.7) in the BGE and SL provided lower limits of detection and greater metabolome coverage than common negative ionization CE–MS methods for metabolic profiling where ammonia containing buffers are used. However, when using the same method in positive ion mode, TEA could lead to ion suppression issues.

**Figure 1:**
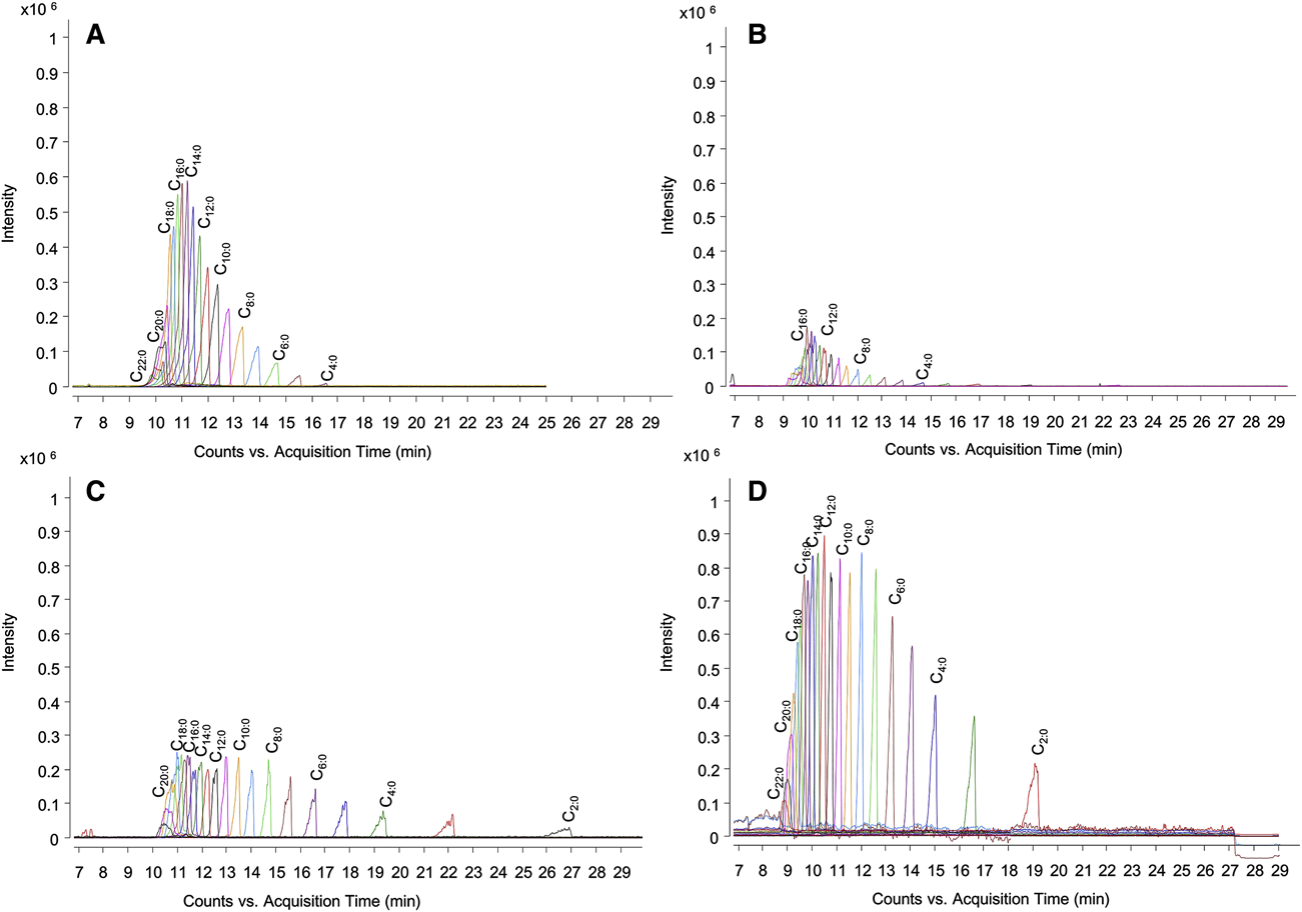
Extracted ion electropherograms of 21 linear FAs (C2∼C22:0, 100 µg/mL) obtained (A) in negative ESI mode by CE–MS using a BGE of 40% acetonitrile in 30 mM ammonium formate at pH 10 (B) in positive ESI mode with a pre‐column technique and adding 250 µL ion pair reagent 1 (IPR1) in the FA standard mixtures, (C) in positive ESI mode with an on‐column technique and adding 250 µL IPR1 into the BGE solution, and (D) in positive ESI mode with a post‐column technique using a SL containing 250 µL IPR1 in 50% IPA solution. Reproduced from 33 with permission.

A few years ago, a novel technique called paired ion electrospray ionization (PIESI) was developed by Armstrong and co‐workers 32. PIESI employs specially synthesized multifunctional cationic ion‐pair reagents (IPRs) to form positively charged adducts with the anions to be analyzed. The adducts are detected in the positive ion mode and at higher *m/z* ratios providing improved S/N and LODs that often are orders of magnitude better than those obtained with native anions in the negative ion mode. Recently, Lee et al. developed a CE‐PIESI‐MS method to analyze fatty acids (FAs) 33. In this study, di‐cationic IPRs were continuously introduced into the SL interface to generate positively charged adducts for anionic metabolites after electrophoretic separation. A preliminary study has shown that the addition of IPR prior to or during separation showed less effective complex formation and thus less improvement in terms of sensitivity compared to negative ion mode detection (Fig. 1). An optimized concentration of 250 µM IPR was added to the SL, as this ensured sufficient complexation without contaminating the ion source. The developed method (BGE: 30 mM ammonium formate in 40% acetonitrile, pH 10) provided LODs which ranged from 0.13 to 2.88 µg/mL for 15 FAs in a test mixture. The CE‐PIESI‐MS method was used to determine FAs in cheddar cheese and powdered coffee samples and showed that regarding sample preparation, in contrast to GC–MS and LC–MS methods, only a simple sample extraction step was needed to measure the FA concentration in the samples without signal suppression.

In order to achieve coverage for a broad range of metabolite classes, analytical approaches are needed that can be used for the profiling of both cationic and anionic metabolites, ideally using the same separation conditions (i.e., capillary, BGE, and SL), and in some cases the same CE polarity and/or MS detection mode, as described by the following examples. In 2010, Wakayama et al. created a separation method for the analysis of amino acids and carboxylic acids, where detection of both species was achieved in a single CE–MS run by changing the polarity of the ESI–MS during the time difference of the detection time between cationic and anionic metabolites 34. When analyzing solely in positive ion mode, amino acids were mainly detected in their protonated form: [M+H]^+^, whereas the anionic carboxylic acids were mostly detected as adduct peaks: [M+NH_4_]^+^. Subsequently, when analyzing the same compound mixture in negative ion mode without adjusting the main parameters, several amino acids were detected in their deprotonated form: [M−H]^−^. In the optimized CE–MS method, the ESI‐MS polarity was changed from positive to negative after the detection of amino acids. A relatively high sheath‐gas pressure (20–25 psi versus 5 to 10 psi in conventional CE–MS experiments) was required for stable spray formation, especially in negative ion mode. The CE–MS method was used to study the Crassulacean acid metabolism in pineapple leaf extracts, and showed effective determination of both anionic and cationic metabolites in a single run. The results confirmed the diurnal change in malate, aspartate, asparagine, and citrate concentration in the pineapple leaves, and showed an opposite pattern for succinate and glutamine.

**Figure 2:**
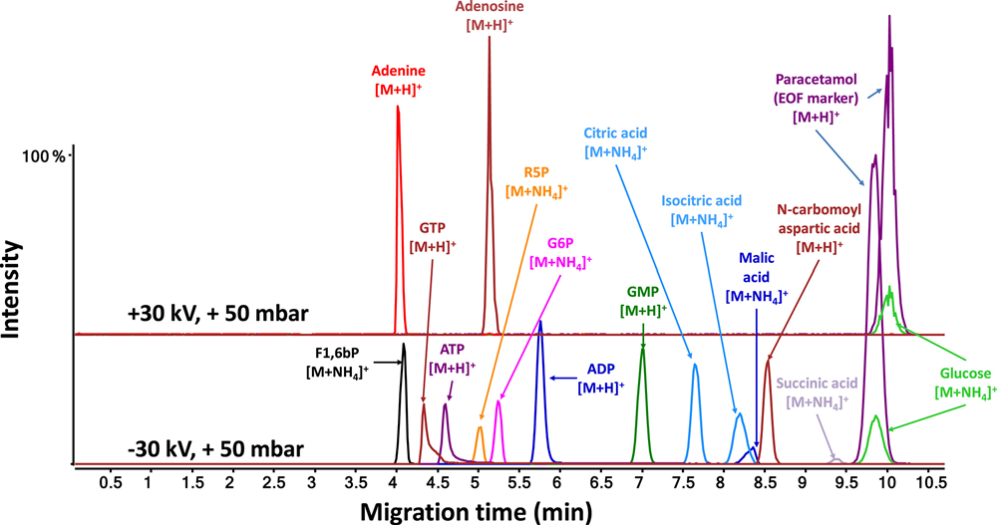
Extracted ion electropherograms obtained for a metabolite test mixture by CE–MS in positive ion mode. Electrophoretic separation performed at low‐pH separation conditions using 10% acetic acid as BGE. Reproduced from 10 with permission.

Drouin et al. developed a CE–MS method for the analysis of both cationic and anionic metabolites in one single run by measuring solely in positive ion mode 10. A two‐step approach was conducted, where cationic metabolites were measured in normal CE polarity, and reverse CE polarity was used to measure anionic metabolites. In agreement with the study performed by Wakayama et al. 34, adduct formation was observed when applying positive ion mode. Moreover, it was found that the formed ammonium adducts for compounds (cations and anions) without amino groups showed higher analyte responses compared to their deprotonated species when detected in negative ion mode. The sensitivity was further enhanced by adjusting some MS source parameters (higher capillary voltage: 5500 V, higher sheath‐gas: 11 L/min) and turning off the nebulizing gas. The CE–MS method could be used for the analysis of both cationic and anionic species in positive ion mode, as shown in Fig. 2 for a test mixture of metabolites. The method was applied to analyze a commercially available metabolomics library, and more than 76% of the 596 compounds could be observed. The obtained findings revealed that CE–MS was especially well‐suited for the analysis of polar and charged metabolites, providing complementary results in comparison to chromatographic‐based techniques. However, it should be noted that this CE–MS method employed a relatively high fragmentor voltage (380 V), which might induce in‐source fragmentation due to energetic collisions that occur in the ESI source. This effect was earlier studied by Godzien et al., who evaluated the impact of the enhancement of fragmentor voltage (150‐230 V) on in‐source fragmentation 35.

Next to the conventional SL interface, new low‐flow SL interfacing designs have been recently developed for CE–MS‐based metabolomics studies. Liu et al. developed a modified co‐axial SL nanosprayer to analyze nucleotides in single neuronal cell extracts 36. The lab‐fabricated nanosprayer has a smaller diameter capillary outlet 20, which is 40 µm instead of a typical 50 or 75 µm internal diameter, allowing lower SL flow rates (<1 µL/min) and no nebulizer gas. These adjustments reduced sample dilution, improved repeatability, and detection limits. In order to withstand corrosion, a platinum alloy emitter was used. Analysis was performed using a BGE of 20 mM ammonium bicarbonate (pH 10) and a SL of isopropanol/water (1:1, v/v) containing 0.2 mM ammonium bicarbonate, which was delivered at 600 nL/min. The method allowed the analysis of nucleotides in extracts of individual *Aplysia californica* sensory neurons with LODs ranging from 2 to 22 nM. The implementation of an on‐line preconcentration method, i.e., large‐volume sample stacking, further improved the detection limits of the method.

**Figure 3:**
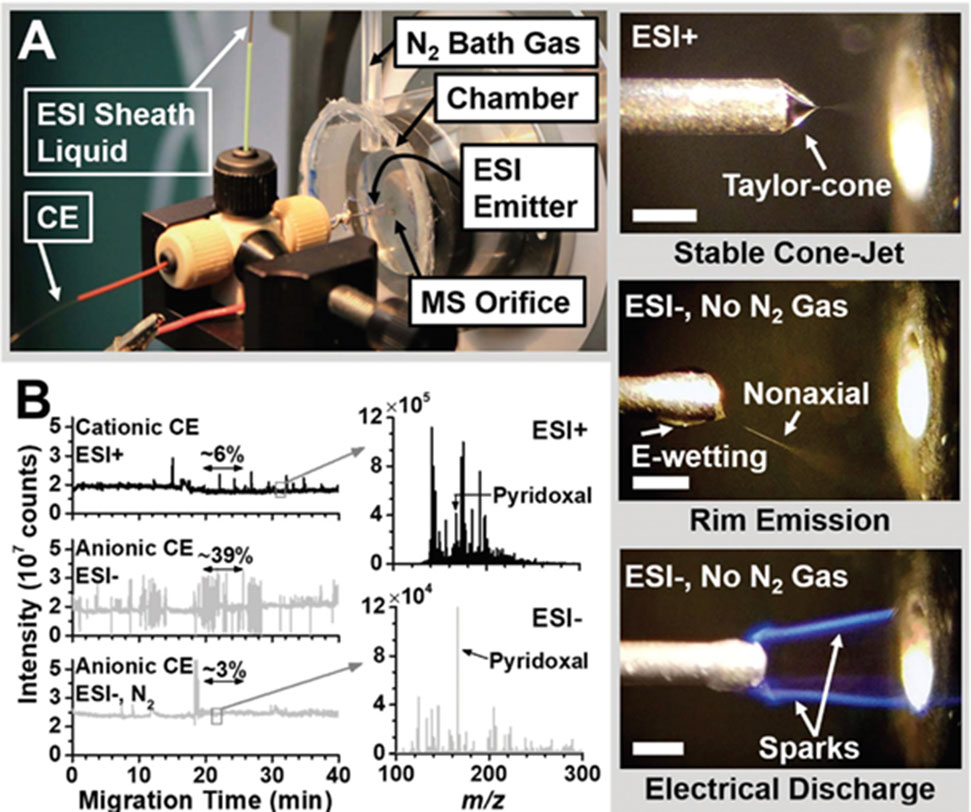
CE–MS for cationic and anionic metabolic profiling. (A) The CE–MS interface with major components labeled. Microscopy comparison of stable Taylor‐cone in ESI+ (top panel) and non‐axial (rim) emission (middle panel) and electrical discharge (spark) in ESI− without nitrogen bath gas. Scale bars = 250 µm. (B) Total ion chromatogram (TIC) revealing stable operation during cationic separation with ESI+ (top panel). ESI in negative ion mode for anionic separation (middle panel) was stabilized upon enclosing the electrospray emitter in a nitrogen‐filled environmental chamber (bottom panel). Spray stability is quantified as percentage RSD of the total ion current. Representative mass spectra of a V1 cell extract revealing simplified spectral complexity during ESI−. Reproduced from 37 with permission.

Recently, Portero et al. developed a method that allows sequential cationic and anionic analysis of metabolites in a single cell off a live vertebrate embryo of the South African frog *Xenopus laevis* 37. The low‐flow SL CE–MS interface was custom‐built and supplemented with a nitrogen gas filled chamber to minimize electrical discharges and produce a stable ESI spray in both positive and negative ion mode. The design of the interface and how the behavior of the electrospray at the ESI emitter tip was evaluated, which included a comparison with the stable Taylor‐cone normally obtained in positive ion mode and with electrical discharge observed in negative ion mode without using the nitrogen gas filled chamber, is shown in Fig. 3A. Additionally, Fig. 3B illustrates the stabilization of the total ion electropherogram signal measured in negative ion mode when including the nitrogen bath gas. The sample was collected via in situ capillary micro‐sampling 38, where circa 10 nL of the cell content (i.e., 5% of the total cell volume) was aspirated. Subsequently, a one‐pot metabolite extraction was applied by ejecting the collected sample into 4 µL mixture of acetonitrile/methanol/water (4:4:2, v/v/v). The resulting cell extract was analyzed by CE–MS using low‐pH separation conditions for cationic metabolites. The separation performance was comparable to other CE–MS methods, such as CE–MS employing a sheathless porous tip interface 39. Overall, the proposed approach is very promising for single cell analysis, although, a single cell with relatively large dimensions (circa 200 nL content) was used in this work. It would be of great interest to develop a CE–MS workflow for single cell mammalian metabolomics, as the content of such cells often range in the low pL‐range (e.g., content of single HepG2 cells corresponds to roughly 3 pL). Clearly, this work is still an enormous analytical challenge.

**Figure 4:**
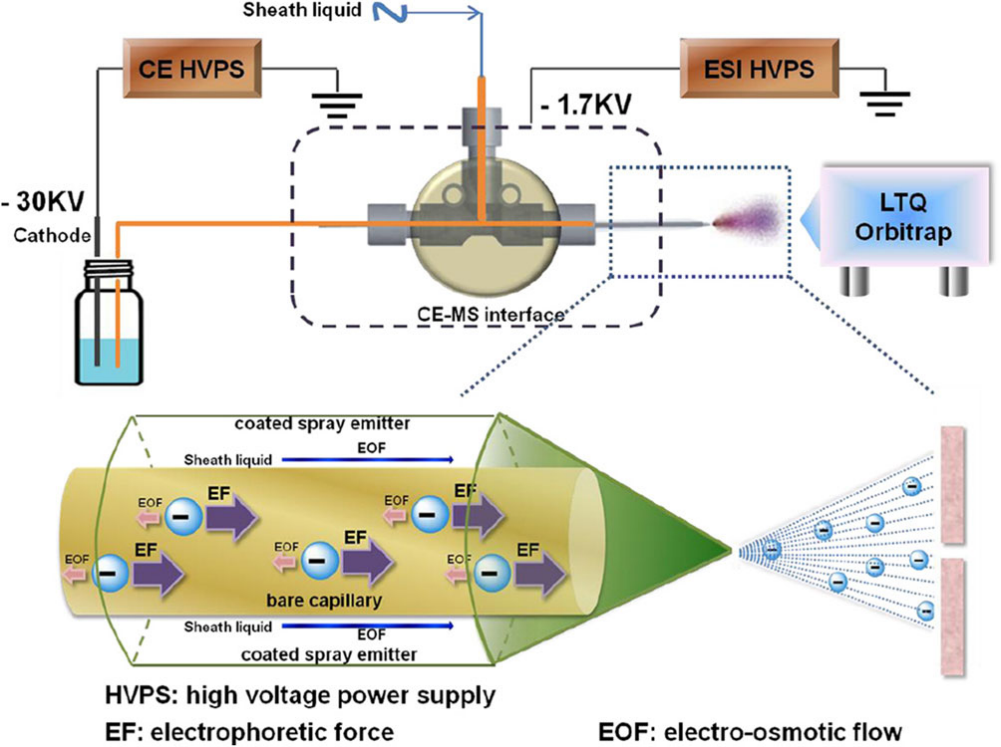
Schematic representation of the negative mode CE–MS system. A reverse polarity separation under a dominant electrophoretic force and low EOF is used to move analytes down a bare separation capillary. The cathode end of the separation capillary is capped with a protein‐coated spray emitter sheath capillary with SL pumped by EOF, mixing with separation flow and affording a stable electrospray of negatively charged analytes that is introduced into an LTQ Orbitrap for MS analysis. Reproduced from 40 with permission.

Lin et al. demonstrated a novel electrokinetically pumped SL interface to analyze heparin oligosaccharides 40. In this method, 10 mM ammonium acetate containing 80% v/v methanol (pH 7.5) was used as BGE and SL, the separation was conducted in reverse CE polarity and negative ion mode conditions. The capillary end was capped with a protein‐coated spray emitter sheath capillary, allowing the addition of SL to the EOF and creating a stable electrospray (Figure 4). The optimized CE–MS method showed to be applicable for disaccharide compositional analysis, bottom‐up analysis, and top‐down analysis. For the top‐down analysis performed with CE–MS, a lower level of ammonium adduct formation was observed than when using HILIC‐MS, resulting in less false positives 41.

CE–MS‐based metabolomics studies are typically performed with a SL interface. However, the addition of SL will cause dilution of the CE effluent, thereby limiting the detection sensitivity. Therefore, new interfacing designs such as the sheathless porous tip interface 42 are gaining interest for metabolomics studies 17, 43, 44. Bonvin et al. developed a non‐aqueous CE–MS (NACE–MS) method for the analysis of acidic compounds using a sheathless interface 45. In NACE, instead of an aqueous BGE, an organic solvent is used as BGE. In this NACE–MS method, the BGE was composed of 5 mM acetic acid in an acetonitrile/methanol mixture (80:20, v/v). The sheathless NACE–MS method improved the sensitivity by 2 to 50fold for the determination of glucuronides in human urine as compared to results obtained with a SL interface.

Gulersonmez et al. evaluated the performance of CE–MS using a sheathless porous tip interface for the analysis of anionic metabolites in biological samples by employing the same experimental conditions as for the profiling of cationic metabolites, only switching the CE separation and MS detection polarity 39. The injection volume was approximately 20 nL and LODs between 10 and 200 nM were obtained for test compounds, showing a significant improvement when compared to LOD values obtained with SL CE–MS systems. It should be noted that this approach can only be applied for the separation of acidic metabolites with a pKa value below 4.2. The limited durability of using a single porous tip capillary (typically up to 200 runs) in CE–MS prevents its use for the analysis of large sets of biological samples 39, 46. A proper sample preparation is critical when using these capillaries. Still, CE–MS with a sheathless interface has shown to have high potential for volume‐restricted metabolomics.

### 2.2 Improving reproducibility

One of the main reasons for the underuse of (standardized) CE–MS methods for metabolic profiling of anionic species is the concern of its lack in reproducibility. In a recent study performed by Acunha et al., a fused‐silica capillary was coated with poly‐(*N,N,N’,N’*)‐tetraethyldiethy‐lenetriamine, *N*‐(2‐hydroxypropyl) methacrylamide (PTH), and its utility for the profiling of anionic metabolites was evaluated in orange juice and wine samples 46. The polymeric dynamic coating could be generated in an automatic procedure and the CE–MS method provided an acceptable repeatability for anionic metabolic profiling, i.e., RSDs (*n* = 3) for migration times and peak areas for selected metabolite standards were below 0.2 and 2.1%, respectively. Additionally, ATP was detected when employing the PTH coating, while it was not detected using an uncoated capillary. Overall, the CE–MS method allowed the detection of 87 metabolites in orange juice and 142 metabolites in red wine, demonstrating the utility of this approach for the characterization of food products.

**Figure 5:**
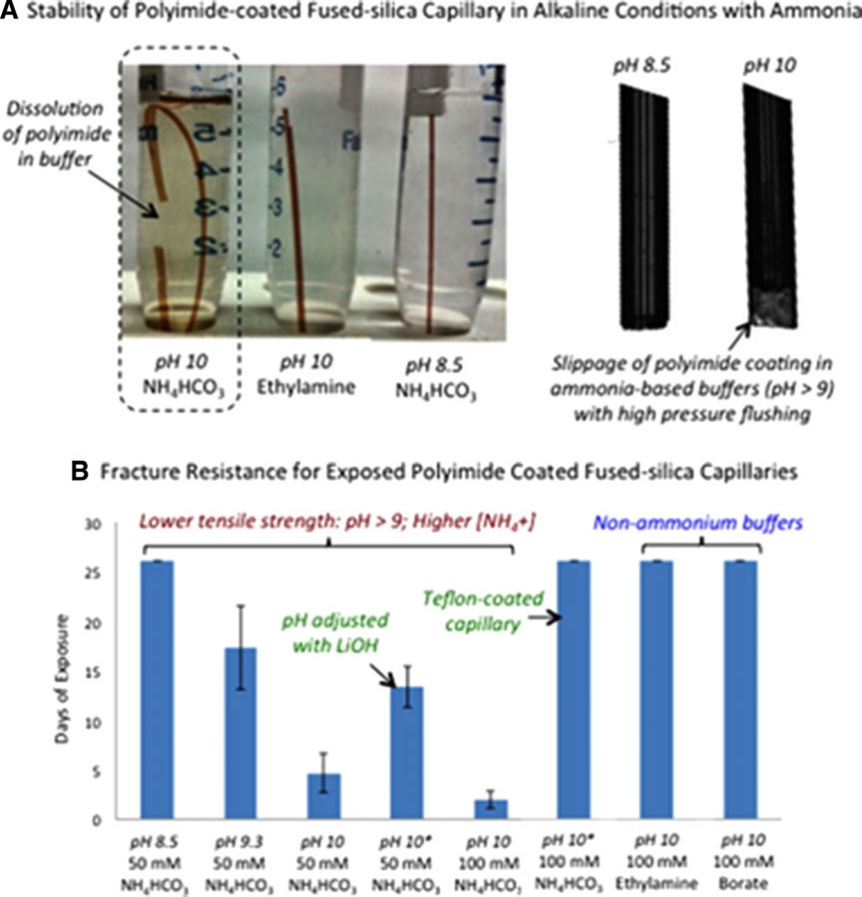
(A) Images showing the impact of prolonged exposure (70 days) of polyimide coated fused‐silica capillaries in aqueous alkaline ammonium buffers that result in softening/deformation of the outer coating and polymer dissolution. High‐pressure (90 kPa) flushing with 50 mM ammonium bicarbonate buffers for 24 h demonstrate elongation of the outer polymer coating beyond the fused‐silica capillary tip at pH 10 that is not observed at pH 8.5. (B) A comparison of changes in the tensile strength and resistance to fractures with repeated bending (90°) to a series of fused‐silica capillary segments (where error bars represent ±1 s, *n* = 6) exposed to different aqueous alkaline solution (i.e., buffer type, pH, ionic strength), indicating that higher ammonia concentrations and increased pH conditions accelerate polyimide aminolysis, shortening their average lifespan due to capillary column breakage. Reproduced from 47 with permission.

Yamamoto et al. has demonstrated that the use of alkaline aqueous ammonia solutions (pH > 9) as BGE leads to chemical degradation of the outer polyimide capillary coating, causing incidental capillary fractures 47. This effect is depicted in Figure 5A, where images of polyimide coated fused‐silica capillaries are shown after an exposure of 70 days to different aqueous alkaline solutions. Long‐term exposure of the capillary to an ammonium bicarbonate solution (pH 10) resulted in degradation of the outer polyimide coating. This was also observed when using other ammonium containing buffers such as ammonium acetate or ammonium hydroxide at elevated pH conditions. When exposing the capillary to aqueous alkaline buffers free of ammonia such as borate (pH 10), or to a primary/secondary amine buffer such as ethylamine (pH 10), no weakening of the capillary coating was observed. Additionally, when using a weakly alkaline ammonium bicarbonate buffer (pH 8.5), there was no capillary degradation. These findings were confirmed when the fracture resistance of fused‐silica capillaries was compared after a bending force (90° angle) was applied manually, where each capillary was exposed to a different solution over a 26‐day period (Figure 5 ). Subsequently, they tested the long‐term performance of the polyimide coated capillaries when using compatible alkaline BGEs, i.e., ammonium bicarbonate (pH 8.5), ammonium acetate (pH 8.5), ethylamine (pH 10.0), diethylamine (pH 11.0), and pyrrolidine (pH 11.3), for profiling anionic metabolites in biological samples. Pooled urine samples were measured in 34 runs (corresponding to 238 repeated sample injections) under alkaline conditions using multi‐segment injection (MSI)‐CE–MS with negative ion mode detection 16. In MSI, multiple samples can be injected serially in a single capillary, in which the sample segments are spatially positioned between BGE zones. A throughput of <5 min per sample was achieved. Hence, aminolysis of the outer polyimide capillary coating can effectively be prevented by using less alkaline ammonium buffers (<pH 8.5) or substituting the BGE by less nucleophilic or alkaline buffers without ammonia. The findings of this work have important consequences as various CE–MS methods described in this paper employed BGEs with a pH above 9.0 and often it was not clear from these papers whether the polyimide coating was removed. Therefore, it is crucial to consider this work in the design of CE–MS methods for anionic metabolic profiling at high‐pH separation conditions.

Large‐scale metabolomics is a promising approach to identify novel biomarkers. Recently, Harada et al. assessed the long‐term performance of CE–MS for metabolic profiling of more than 8000 human plasma samples from the Tsuruoka Metabolomics Cohort Study over a 52‐month period 48. Using two separate CE–MS systems for anionic and cationic metabolic profiling, respectively, more than 150 polar metabolites could be identified. The study provided an absolute quantification for 94 polar metabolites in plasma with a similar or better reproducibility (i.e., in QC samples RSD for peak area was below 20% for 64 metabolites and less than 30% for 80 metabolites out of the 94 metabolites) when compared to other MS‐based analytical platforms employed for large‐scale metabolomics studies. The overall metabolic coverage of the employed CE–MS methods was limited in comparison to other established LC–MS platforms, as CE–MS was not able to detect most of the non‐polar metabolites in plasma. Azab et al. developed a standardized high‐throughput NACE–MS method for the determination of FAs in blood specimens 49. A MSI approach was used where a serial injection of seven independent samples (including a quality control sample) within a single CE run was applied, thereby improving the throughput of the method. For the separation, a BGE of 70% ACN, 15% MeOH, 10% H2O, and 5% isopropanol in ammonium acetate (pH 9.5), and a SL of 80% MeOH with 0.5% ammonium hydroxide was used. In order to prevent aminolysis and swelling of the outer polyamide capillary coating which is often the case when using alkaline ammonia containing BGEs (pH > 9.0) 47 and organic solvents that have long‐lasting contact with the capillary 50, the outer coating was removed from the capillary terminal ends (±7 mm). During capillary flushing and sample injection, the nebulizer gas was turned off to prevent causing current drops caused by air plugs inside the capillary. In order to prevent corona discharge in negative ion mode, a sprayer voltage of −3.5 kV was used. In the optimized MSI–NACE–MS method, seven serum extracts were analyzed after a methyl‐tert‐butyl ether extraction 51 without chemical derivatization, and most FAs were detected as their deprotonated molecular ions [M–H]^−^. A throughput of <4 min/sample was achieved, and the method sensitivity showed to be comparable to conventional GC methods. This work clearly shows the utility for MSI–NACE–MS for lipid profiling.

Recently, Höcker et al. assessed the analytical performance of CE–MS for a selected group of analytes using three different interfacing designs, i.e., the co‐axial SL interface, the electrokinetic‐driven nanoflow SL interface, and the sheathless porous‐tip interface 52. The conventional SL interface showed good robustness and flexibility, but in case an improved detection sensitivity was required, both the electrokinetic‐driven SL interface and sheathless interface could be considered for this purpose, as they provided a sensitivity enhancement of an at least 100fold. Still, in contrast to CE–MS using a standard SL interface, the long‐term performance of the latter interfaces still needs to be demonstrated. The durability or lifespan of a single capillary in these interfaces is also an important aspect and such data needs to be provided, preferably by multiple laboratories. It should be noted that in this study, a standard SL interface was employed. Recent studies have shown that with alteration of the MS source parameters, such as for example setting the nebulizing gas to 0 psi, a sensitivity improvement of an at least 15fold can be achieved using a SL interface 10.

## 3 Conclusions and perspectives

CE–MS employing a standard SL interface performs well for cationic metabolic profiling as shown by various research groups. However, concerning anionic metabolic profiling, further development is needed in order to obtain a reliable approach that can preferably be easily used by multiple groups. One of the main challenges is the search for the most optimal CE–MS interfacing design when it comes to robustness, sensitivity, and user‐friendliness. Overall, on the basis of our assessment of the reported literature in this specific field in the given time period, the development of a highly sensitive and reliable CE–MS method for anionic metabolic profiling will remain an active area of research. In our opinion, the profiling of anionic metabolites in limited amounts of mammalian cells will be an important application field of CE–MS. The implementation of rigorous validation and the availability of standard operating procedures would be highly favorable in order to make CE–MS an alternative, viable analytical technique for metabolomics. Therefore, our intention is to set‐up an inter‐laboratory CE–MS study using both the standard SL and the sheathless porous tip interface for metabolic profiling. Such data is urgently needed to actually show the suitability of CE–MS for long‐term anionic metabolic profiling. The availability of open access peer‐reviewed protocols would be very helpful for this purpose, and in that context the developments are going into the right direction 38, 53.

*The authors have declared no conflict of interest*
